# Predicting Amyotrophic Lateral Sclerosis Mortality With Machine Learning in Diverse Patient Databases

**DOI:** 10.1002/mus.28487

**Published:** 2025-07-28

**Authors:** Ling Guo, Ian Qian Xu, Sonakshi Nag, Jing Xu, Josiah Chai, Zachary Simmons, Savitha Ramasamy, Crystal Jing Jing Yeo

**Affiliations:** ^1^ Institute for Infocomm Research (I^2^R) Agency for Science, Technology and Research (A*STAR) Singapore; ^2^ Duke‐NUS Medical School Singapore; ^3^ National Neuroscience Institute Singapore; ^4^ Centre for Quantitative Medicine, Duke‐NUS Singapore; ^5^ Department of Neurology Pennsylvania State University Hershey Pennsylvania USA; ^6^ Institute of Molecular and Cell Biology Agency for Science, Technology and Research (A*STAR) Singapore; ^7^ School of Medicine, Medical Sciences and Nutrition University of Aberdeen Aberdeen UK

**Keywords:** amyotrophic lateral sclerosis, diverse external validation, machine learning, mortality prediction, survival analysis

## Abstract

**Introduction:**

Predicting mortality in Amyotrophic Lateral Sclerosis (ALS) guides personalized care and clinical trial optimization. Existing statistical and machine learning models often rely on baseline or diagnosis visit data, assume fixed predictor‐survival relationships, lack validation in non‐Western populations, and depend on features like genetic tests and imaging not routinely available. This study developed ALS mortality prediction models that address these limitations.

**Methods:**

We trained Royston‐Parmar and eXtreme Gradient Boosting models on the PRO‐ACT database for 6‐ and 12‐month mortality predictions. Each visit was labeled positive (for death) if death occurred within 6 or 12 months, negative if survival was confirmed beyond that, and excluded if follow‐up was insufficient, assuming patients were alive up to their last recorded visit. Models were validated on independent datasets from the North American Celecoxib trial and a Singapore ALS clinic population. Feature importance and the impact of reducing predictors on performance were evaluated.

**Results:**

Models predicted mortality from any clinical visit with area under the curve (AUC) of 0.768–0.819, rising to 0.865 for 12‐month prediction using 3‐month windows. Albumin was the top predictor, reflecting nutritional and inflammatory status. Other key predictors included ALS Functional Rating Scale‐Revised slope, limb onset, absolute basophil count, forced vital capacity, bicarbonate, body mass index, and respiratory rate. Models maintained robust performance on the independent datasets and after reducing inputs to seven key predictors.

**Discussion:**

These visit‐agnostic models, validated across diverse populations, identify key prognostic features and demonstrate the potential of predictive modeling to enhance ALS care and trial design.

**Abbreviations**
ALS: amyotrophic lateral sclerosis
ALSFRS‐R: ALS functional rating scale‐revised
AUC: Area under the receiver operating characteristic curve
AUPRC: area under the precision‐recall curve
BiPAP: Bilevel positive airway pressure
BMI: body mass index
CI: confidence interval
CIRB: centralized institutional review board
DTUA: data transfer and use agreement
ENCALS: European network to cure ALS
FVC: forced vital capacity
KNN: k‐nearest neighbor
ML: machine learning
MRI: magnetic resonance imaging
NEALS: northeast ALS consortium
NIV: noninvasive ventilatory support
NPV: negative predictive value
PCC: Pearson correlation coefficient
PPV: positive predictive value
PRO‐ACT: pooled resource open‐access ALS clinical trials
RP: Royston‐Parmar
SD: standard deviation
XGBoost: eXtreme gradient boosting
SG ALS: Singapore ALS database

## 1 Introduction

Amyotrophic Lateral Sclerosis (ALS) exhibits widely varying rates of functional decline and survival across patients and regions [1, 2]. This variability complicates clinical care, confounds power calculations in trials, and delays therapeutic advances. Accurately predicting survival can improve patient management, guide personalized palliative care and end‐of‐life decisions, and permit better selection of patients for clinical trials [3, 4, 5, 6]. For example, depending on the trial objective and intervention type, selecting early‐stage ALS patients with shorter life expectancy can shorten trial duration, reduce costs, and increase the chances of observing significant treatment effects [6].

Early prognostic efforts focused on traditional statistical models, such as Cox proportional hazards or parametric survival models [7, 8, 9, 10]. Lunetta et al. [10] devised an “ALS Survival Score” by weighting six baseline factors in a multivariate Cox model, achieving an area under the receiver operator characteristics curve (AUC) of 0.75 on an independent dataset. Elamin et al. [8] combined diagnostic delay, onset site, age, and forced vital capacity (FVC) into a simple risk algorithm, obtaining positive predictive value (PPV) of 73.3%–85.7% and negative predictive value (NPV) of 93.3%–100%. The European Network to Cure ALS (ENCALS) model [11] extended these ideas using a Royston‐Parmar (RP) flexible‐parametric framework with eight baseline variables with AUC of 0.78–0.86. However, it requires data from the diagnosis visit specifically, which may not be readily available and ignores critical disease progression information from subsequent clinical visits. These traditional models also make strong assumptions about data (e.g., proportional hazards, that is, assuming the effect of a risk factor on survival is constant over time) and struggle with missing data, complex non‐linear variable interactions, and heterogeneity in clinical datasets.

To address these limitations, non‐parametric machine learning (ML) methods, which do not assume a fixed mathematical relationship and can adapt to more complex patterns in the data, have been applied [12]. Ong et al. [13] applied AdaBoost to five longitudinal laboratory measures, obtaining an AUC of 0.77. Pfohl et al. [14] used random forests on 38 clinical and functional variables and achieved an AUC of 0.77–0.907, with performance that improved with longer mortality prediction periods. ML models that incorporate magnetic resonance imaging (MRI) have also shown strong discrimination [15, 16]. These models underscore the promise of ML in ALS survival prediction, particularly in handling high‐dimensional heterogenous data.

Nevertheless, key gaps persist. Firstly, patients often make multiple clinical visits, but single‐visit prediction models do not utilize this additional information and account for ALS progression variability [17, 18], potentially affecting prediction accuracy. Secondly, many studies utilized features like genetic testing and MRI data [11, 15, 16, 19], which are difficult and expensive to obtain and process. In contrast, laboratory tests are frequently performed as part of routine care and can be useful for prediction [13, 20]. Lastly, previous studies often lack external validation, making it difficult to assess model generalizability [13]. Most relied on Western datasets [11, 13], with limited focus on Asian patient populations [21]. Although previous studies in Chinese patients identified prognostic factors, they did not develop predictive models and were limited to the Chinese population [20, 22]. Our study addresses these gaps by developing visit‐agnostic, ML‐based ALS mortality prediction models that incorporate any single clinical encounter or aggregated data over 2‐ or 3‐month windows. We utilize routinely collected clinical and laboratory features and evaluate external validity on both a North American trial dataset [23] and an Asian ALS cohort from Singapore [24].

We selected the eXtreme Gradient Boosting (XGBoost) [25] ML model for its ability to handle complex, real‐world clinical data more effectively than other non‐parametric methods [13, 17, 24, 26, 27]. While tree‐based models have been applied to ALS survival prediction, the use of XGBoost remains limited [13, 14, 28]. XGBoost builds decision trees sequentially, with each tree correcting errors from the previous one, allowing it to model non‐linear relationships and feature interactions without assuming a fixed data distribution. Unlike parametric models, XGBoost tolerates missing values, multicollinearity, and noisy inputs, all common in clinical datasets. It also generates clear feature‐importance scores, highlighting both established and novel prognostic factors. In comparison to prior ML used [13, 14, 15], XGBoost offers a superior balance of accuracy, interpretability, and ease of deployment in clinical settings.

Our objectives are:

1. To develop XGBoost models to predict mortality from any clinical visit, and compare them to RP models used by ENCALS
2. To examine how varying observation windows affect prediction performance
3. To evaluate the impact of incorporating additional features, especially laboratory data, on prediction accuracy
4. To evaluate the model's generalizability across diverse datasets, including data from the Asian patients in Singapore.

## 2 Methods

### 2.1 Data Sources

We used the Pooled Resource Open‐Access ALS Clinical Trials (PRO‐ACT) database [29], a publicly available dataset comprising 10,723 patients from 16 ALS clinical trials and 1 observational study to train and test our models. After excluding patients without mortality data and visits with > 50% missing lab values, the final dataset included 24,020 visits from 1941 patients (Table S1, Figure S1).

The Celecoxib trial [23] and the Singapore (SG) ALS [24] databases were used as additional validation datasets. The Celecoxib trial database (DTUA2021A009305 with Massachusetts General Hospital/Harvard Medical School and the NEALS consortium) is a United States double‐blind, placebo‐controlled, clinical trial of celecoxib (800 mg/day) in ALS patients, consisting of 2662 records from 300 patients. After excluding patients without survival data, 915 visits from 268 unique patients remained (Table S2). These patients were distinct from those in PRO‐ACT used for training and testing the models.

The SG ALS Database comprised 1312 visit records from 72 ALS patients diagnosed at Tan Tock Seng Hospital Singapore, from January 2010 to November 2023 (Table S3). The collection and analysis of the SG ALS database were approved by the Centralized Institutional Review Board at SingHealth (CIRB Ref: 2015/2030) and informed consent was obtained. Feature distribution differences among the three datasets are shown in Table S4. Patients and the public were not involved during the design, conduct, reporting, interpretation, or dissemination of the study.

### 2.2 Machine Learning Models

We implemented two modeling approaches: the parametric RP survival model and the non‐parametric XGBoost algorithm.

The RP model uses a smooth mathematical curve (spline‐based functions with four knots) to estimate how different factors, like age and ALSFRS‐R slope, affect a patient's survival time. It assumes that these factors influence survival in a gradual, consistent way. RP modeling was implemented in R v4.1.2 [30] (R Foundation for Statistical Computing, Vienna, Austria) using the *flexsurv* package.

XGBoost [25] was described earlier in the introduction and was implemented using the python package *xgboost*. XGBoost can capture complex, nonlinear patterns and interactions (e.g., how a combination of low albumin and rapidly declining ALSFRS‐R amplifies mortality risk without predefining that relationship). It has been applied in neurology, for example, post‐stroke outcome prediction based on clinical and imaging data [31, 32].

Inputs to both models were formatted as tabular datasets, with each row representing a single clinical visit or an observation period, and columns representing features (e.g., laboratory values).

We implemented 5‐fold cross‐validation to train and test both models, evaluating performance using AUC, Area Under the Precision Recall Curve (AUPRC), accuracy, sensitivity, specificity, F1 score, PPV, and NPV. AUC represents the model's ability to rank patients who died higher in risk than those who survived. It is computed by comparing all possible pairs of patients (one who died and one who survived) and calculating the proportion of times the model assigns a higher risk score to the patient who died. An AUC of 0.5 reflects random chance, while an AUC of 1.0 indicates perfect separation. Unlike accuracy, AUC, sensitivity, and specificity are not affected by class imbalance.

For RP models, binary predictions (death within 6/12 months) were derived by thresholding the survival probability outputs at the point that maximizes the sum of sensitivity and specificity in the training dataset.

### 2.3 Feature Importance

We assessed feature importance in XGBoost using the gain metric, which quantifies how often and effectively a feature improves model accuracy when used to split decision trees. The features were ranked by the total gain and frequency in the top 15 across 5 cross‐validation runs for both 6‐ and 12‐month mortality. For the RP model, importance was based on standardized coefficients (coefficient magnitude divided by the feature's standard deviation), averaged across folds for both time points.

Because XGBoost combines features in complex, nonlinear ways, the exact relationship between top features and mortality is unclear. To clarify this, we calculated Pearson correlation coefficients (PCC) between individual features and survival duration, treating each clinical visit as a separate data point. PCC values range from −1 (perfect negative) to 1 (perfect positive linear relationship), with 0 meaning no relationship. Only visits with known death dates and non‐imputed values were included.

### 2.4 Data Processing and Study Procedures

The PRO‐ACT database was preprocessed as in our previous study [27]. Missing values were imputed using a two‐step approach: (1) linear interpolation across a patient's visits if data from other visits from the patient is available, (2) if data for individual patients were insufficient, k‐nearest neighbor (KNN) based on age, sex, site of onset, and time since onset. KNN estimates missing values based on the average or majority vote values of k most similar patients. For the test set, KNN imputation was applied using only the training data, ensuring no information leakage. To evaluate real‐world feasibility, we repeated model evaluation using KNN‐only imputation for the test set, which excludes the use of future visits.

For each visit, mortality labels were assigned based on the time from that visit to the patient's recorded date of death. A visit was labeled as positive for 6‐ or 12‐month mortality if death occurred within that time window. Visits were excluded if survival status within the window was unknown due to censoring. Patients without a recorded death date were assumed to be alive up to their last clinical visit, with no assumptions made beyond that point.

We trained single‐visit models, using data from individual visits to predict mortality in the next 6/12 months. We first used the five variables from the ENCALS model that were present in the PRO‐ACT database (age at onset, FVC, diagnostic delay, ALSFRS‐R slope, and bulbar onset). Diagnostic certainty, frontotemporal dementia, and genetic testing (C9orf72 repeat expansion) information were unavailable in the PRO‐ACT database and hence excluded. ENCALS features were processed following the original model specifications to allow direct comparison. Next, we expanded the input to include 48 PRO‐ACT features with sufficient prevalence to assess whether broader clinical data improved performance.

We also investigated the impact of incorporating longitudinal information by comparing models trained on single visits, 2‐ and 3‐month observation windows. Visits were aggregated within each window (mean of 3.0 and 3.8 visits respectively for 2 and 3 months), with mean, standard deviation, and slope (monthly change) computed for each feature. To ensure equal sample sizes across observation periods and avoid bias, we subsampled the training data by randomly selecting the same minimum number of visits per observation period for each model.

To improve model usability, we identified the top 15 features based on XGBoost importance scores and assessed model performance while sequentially reducing features from top 15 to top 5 (i.e., top 15, 14 and so on until top 5). This helped determine the trade‐off between model simplicity and predictive performance.

Finally, we evaluated the XGBoost and RP models, trained on PRO‐ACT, on the SG ALS and Celecoxib trial datasets using [1] the full feature set and [2] reduced sets (top 15 to top 5 features) to assess robustness and risk of overfitting.

## 3 Results

### 3.1 XGBoost and RP models can predict 6‐ and 12‐month mortality based on a single clinical visit

**FIGURE 1:**
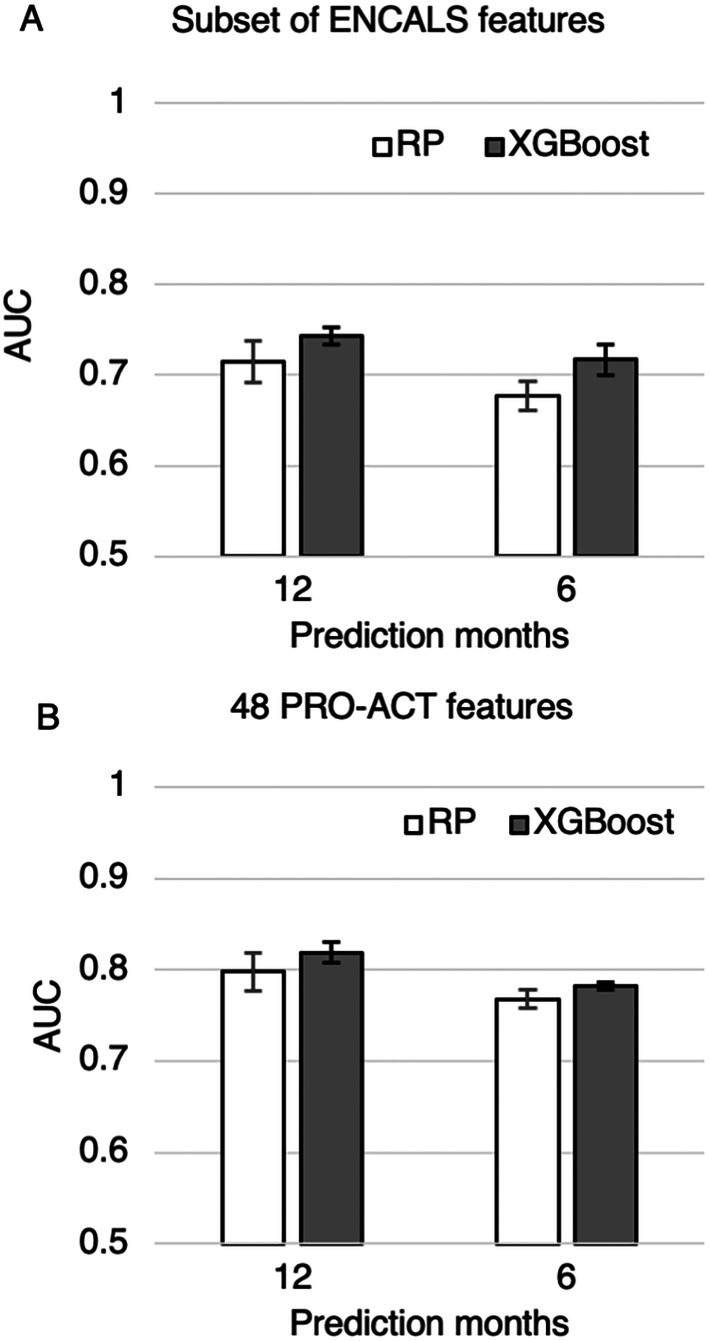
Mortality prediction using (A) subset of ENCALS features present in the PROACT database: FVC, diagnostic delay, age of onset, ALSFRS‐R slope, (B) 48 features present in the PROACT database.

XGBoost and RP models obtained AUCs of 0.677–0.743 (Figure 1A, Table S5), with XGBoost performing better for mortality prediction from a single visit using the 5 ENCALS features present in the PRO‐ACT database.

Using a larger set of 48 PRO‐ACT features, including laboratory test results, improved the performance of both RP and XGBoost models (AUC of 0.768–0.819, Figure 1B, Table S6). XGBoost performed slightly better than RP models. XGBoost maintained strong performance when evaluated on test data using KNN‐only imputation. AUC decreased slightly from 0.819 ± 0.011 to 0.795 ± 0.016 for 12‐month mortality and from 0.782 ± 0.004 to 0.762 ± 0.011 for 6‐month mortality (Figure S2).

### 3.2 Albumin Is a Top Predictive Feature

We identified the top 15 important features for the XGBoost and RP model predictions within the PRO‐ACT database (Tables 1 and S7, Figure S3), which include laboratory test results, disease history, and demographics. Albumin is the top predictive feature for the XGBoost (Table 1) and the 3rd most important for RP (Table S7).

**TABLE 1:** Top 15 features important for 12 and 6‐month mortality prediction for XGBoost models and their association with mortality risk.

Rank	Feature	Association with mortality risk (higher/lower risk)
1	Albumin	Lower
2	Limb onset	Lower
3	Negative ALSFRS‐R slope	Higher
4	Bicarbonate	Higher
5	Absolute basophil	Lower
6	Forced vital capacity	Lower
7	Protein	Lower
8	ALSFRS‐R	Lower
9	Body mass index	Lower
10	Absolute eosinophil	Higher
11	Respiratory rate	Higher
12	Phosphorus	Higher
13	Age	Higher
14	Chloride	Lower
15	Total bilirubin	Higher

*Note*: ‘Higher’ indicates that larger feature values are linked to higher likelihood of death, ‘lower’ indicates that larger feature values are linked to better survival.

Abbreviation: ALSFRS‐*R*, amyotrophic lateral sclerosis functional rating scale‐revised.

### 3.3 Longer Observation Windows Improve Performance for 12‐Month Mortality Prediction

**FIGURE 2:**
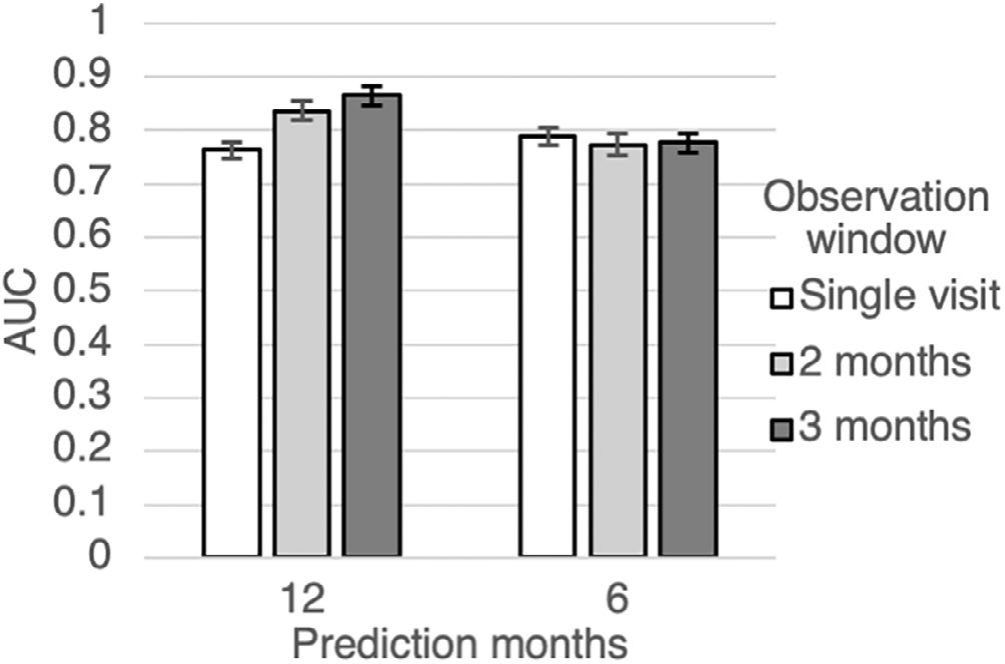
Mortality prediction performance using XGBoost and various observation window lengths.

Longer observation windows are better at predicting 12‐month mortality (0.763 AUC for single visit compared to 0.865 AUC for 3 months observation window, Figure 2, Table S8). However, 6‐month mortality prediction performance is similar for different observation windows.

**FIGURE 3:**
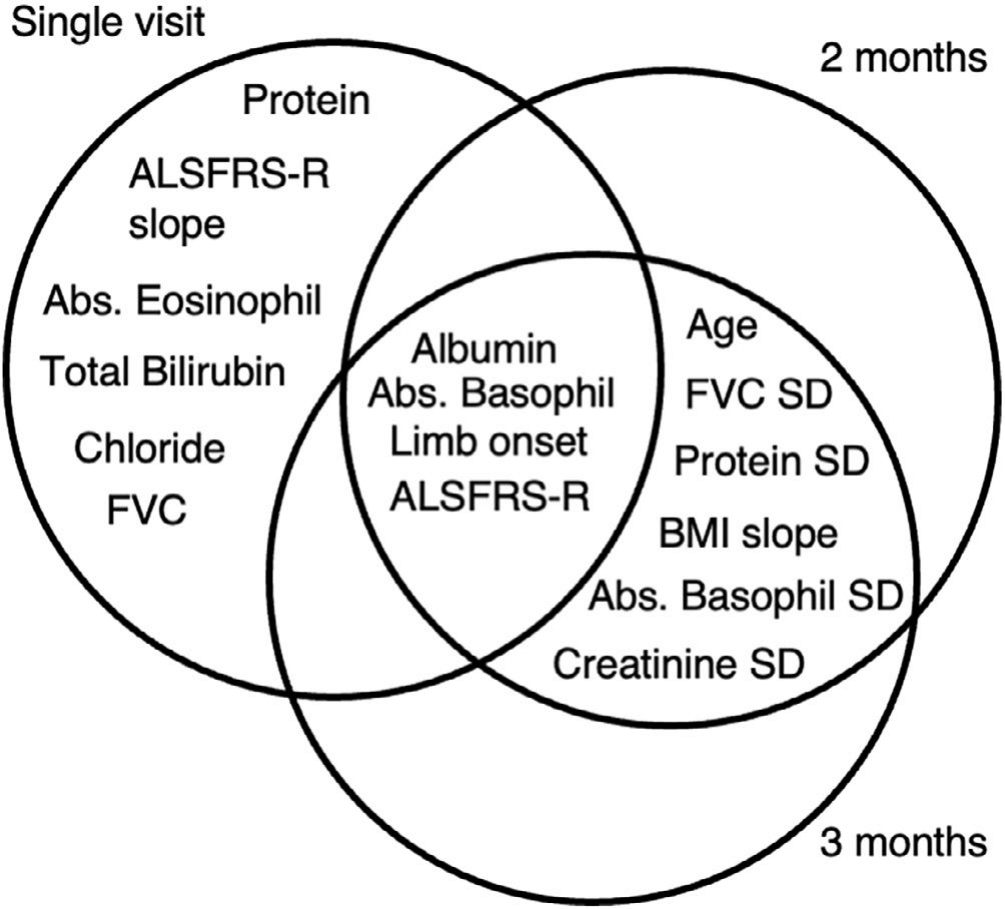
Top important features for mortality prediction using different observation window lengths, for both 6 and 12‐month mortality.

We compared the top predictive features for the XGBoost models trained on various observation windows and identified a consistent set of core predictors: albumin, absolute basophil counts, limb onset, and ALSFRS‐R (Figure 3).

### 3.4 Top Features Used by Machine Learning Models Are Linearly Correlated With Number of Survival Days

The top 10 features are categorized into clinical features, inflammation, nutrition, or respiratory‐related. All the features have significant linear correlations with the number of remaining survival days (Table 2).

**TABLE 2:** Linear correlation between features and survival days in the PRO‐ACT database.

Category	Feature	Correlation with survival days	*p*
Clinical features	ALSFRS‐R slope	0.204	< 0.001
	ALSFRS‐R	0.340	< 0.001
Inflammation	Absolute basophil	0.125	< 0.001
	Absolute eosinophil	−0.119	< 0.001
Inflammation/Nutrition	Albumin	0.235	< 0.001
Nutrition	Protein	0.083	< 0.001
	Body mass index	0.224	< 0.001
Respiratory	Bicarbonate	−0.282	< 0.001
	Forced vital capacity	0.386	< 0.001

Abbreviation: ALSFRS‐*R*, amyotrophic lateral sclerosis functional rating scale‐revised.

### 3.5 Mortality Prediction Performance Is Similar for Mildly and Severely Affected Patients

**FIGURE 4:**
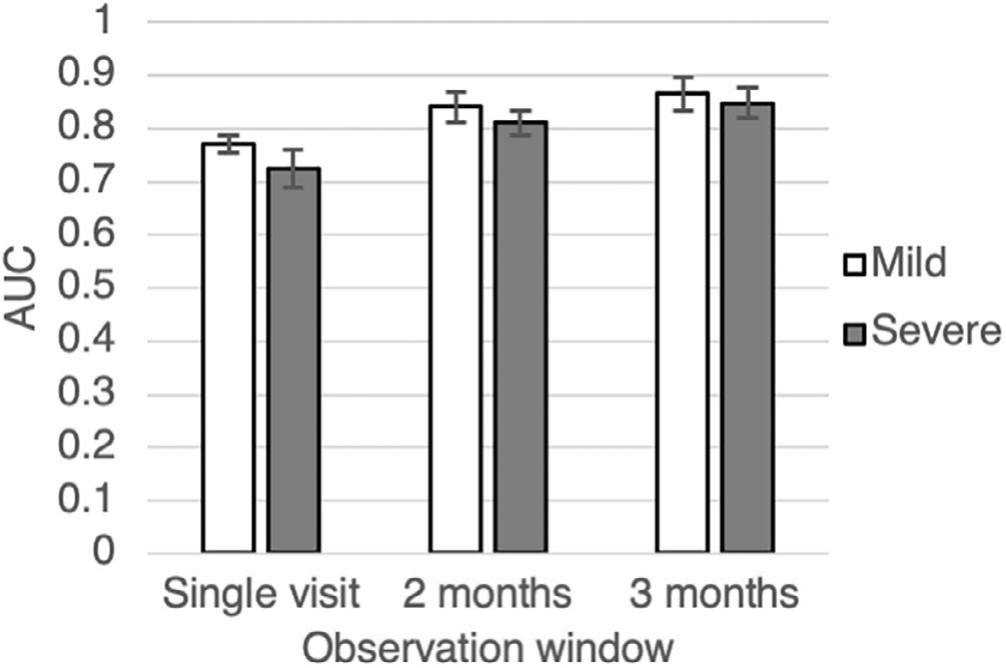
One‐year mortality prediction performance for mildly and severely affected patients.

As ALS has a non‐linear progression [18, 33, 34], we assessed if the models can predict mortality equally well in mildly (mean ALSFRS‐*R* > 30) and more severely (mean ALSFRS‐*R* ≤ 30) affected patients [35]. The XGBoost model has a high AUC of at least 0.725 for predicting mortality across both patient groups for all observation time periods (Figure 4, Table S9).

### 3.6 Input Feature Set Can Be Reduced to 7 Top Predictors With Minimal Loss in Performance

When reducing the number of features progressively from the top 15 to the top 5 predictors, we observed a slight decrease in the Area Under the Curve (AUC) for both models (Figure S4), with the most prominent decrease after 7 features. Hence, using the top 7 most important features provides a good balance between model performance and the number of features. Both the XGBoost and RP models can predict 6‐and 12‐month mortality using the top 7 features with AUC higher or equal to 0.719, with XGBoost outperforming the RP models (Table S10).

### 3.7 Both RP and XGBoost Mortality Prediction Models Generalize Well on External Datasets

**FIGURE 5:**
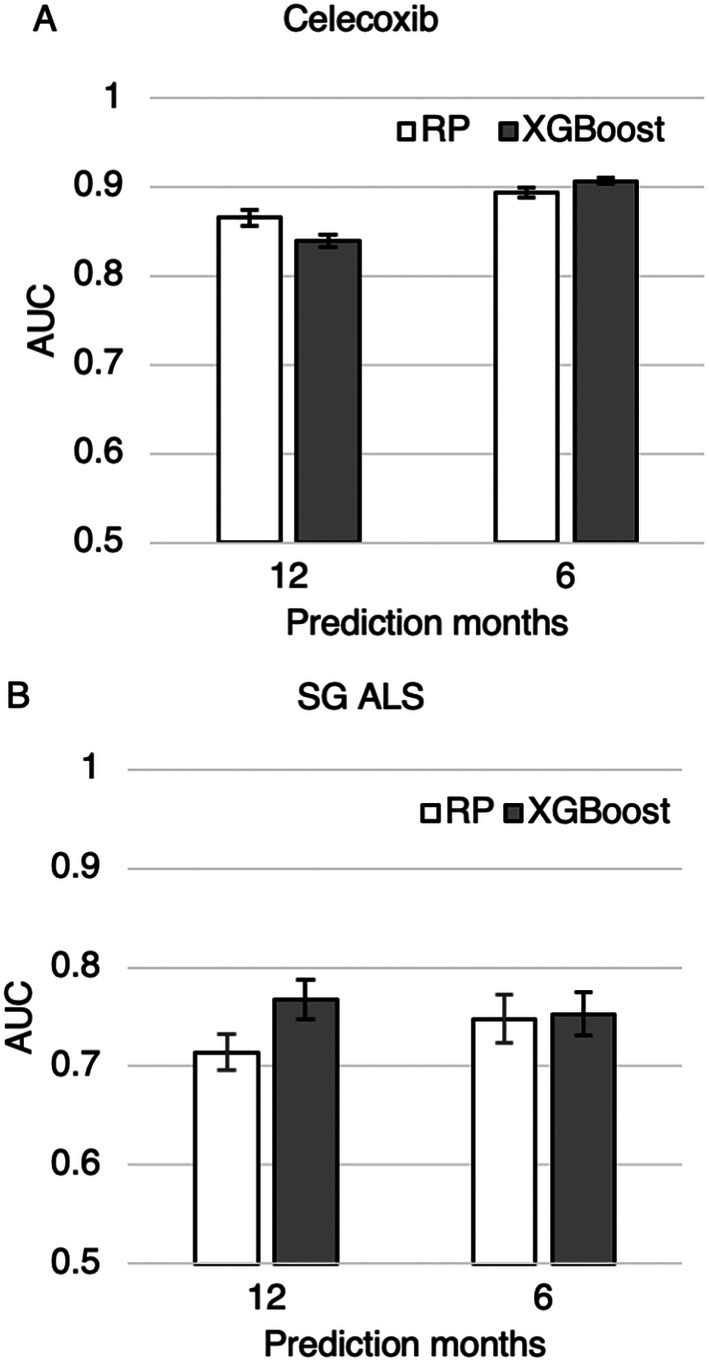
Mortality prediction on the (A) Celecoxib trial dataset and (B) SG ALS dataset using 48 features from the PRO‐ACT database.

We tested the generalizability of the XGBoost and RP models by evaluating the trained models on the North American Celecoxib trial dataset and the SG ALS dataset. Both models generalize well on both datasets, achieving an AUC of at least 0.714 (Figure 5, Tables S11 and S12) using all the PRO‐ACT features and at least 0.681 using the top 7 identified features (Tables S13 and S14).

## 4 Discussion

### 4.1 A Set of Flexible and Practical Models for ALS Mortality Prediction

We developed machine learning models that can predict 6‐and 12‐month mortality from any clinical visit with AUCs of 0.768–0.819, rising to 0.865 when aggregating over a 3‐month observation window. This performance is comparable to prior models, including ENCALS (AUC 0.78–0.86) [11], Ong et al. [13] (0.77), Huang et al. [36] (0.70) and Pfohl et al. [14] (0.85 for 1‐year mortality). Models incorporating MRI data performed well but require imaging that is seldom obtained in routine practice [15, 16]. Models based solely on clinical data rarely exceed AUCs of 0.9. An AUC of 0.8 indicates that the model correctly ranks a patient who dies above one who survives 80% of the time. Our model does not replace clinical judgment but complements it by providing probabilistic estimates that capture nonlinear relationships across multiple features. Even if imperfect, it can still add value by supporting individualized care planning, prompting earlier palliative referrals, identifying trial candidates at different disease stages, and aiding shared decision‐making as a consistent, data‐driven second opinion.

Compared to models restricted to the baseline or diagnosis visit [8, 11, 13], our approach attains similar performance while permitting prediction at any point in the disease trajectory, using a single visit or aggregated data over 2–3 months. This visit‐agnostic property allows users to make use of readily available and current information for mortality prediction [11, 13, 36]. Notably, aggregating data improves 12‐month mortality prediction, but for 6‐month predictions, a single recent visit is often sufficient, likely because imminent mortality is already signaled by markers in the latest visit. Clinically, this means that ALS clinics and trialists can use our single‐visit model to triage patients quickly, then refine risk estimates if additional visits accrue before enrollment or intervention. Importantly, our models performed well in both early‐ and late‐stage ALS patients, confirming their utility across the disease spectrum.

Unlike models requiring MRI [15, 16] or genetic testing data [11], which may be unavailable in routine or resource‐limited settings, our models use more commonly available demographic, clinical, and laboratory data. This makes our models more accessible and practical for use in a wider group of patients and a wider range of practice settings. We also showed that predictive accuracy is preserved with as few as 7 input features, improving usability and offering a practical alternative when full data are not available. While using even fewer features (e.g., top 3) is appealing, the performance drop will be more substantial. Ultra‐simplified models may be considered in future work aimed at point‐of‐care deployment or mobile health applications.

Finally, by validating our models on both the North‐American Celecoxib trial and an Asian tertiary‐care cohort, we confirmed their generalizability across ethnically and geographically distinct populations, an attribute rarely quantified in earlier studies. The two datasets differ in (1) ethnicity and (2) the Singapore database represents a clinic population, whereas the Celecoxib dataset, like PROACT, is from clinical trials. These results not only highlight the models' robustness across different populations and datasets but also their potential applicability in varied clinical and research settings.

### 4.2 Comparison of RP and XGBoost Models

In the PRO‐ACT dataset, XGBoost consistently outperformed the RP model in predicting ALS mortality, both when using ENCALS‐selected features and the full PRO‐ACT feature set. This advantage remained even as the feature set was reduced from the top 15 to the top 5 features, highlighting XGBoost's ability to capture complex, nonlinear relationships, even within compact input sets. In contrast, performance between XGBoost and RP was similar on the external Celecoxib and SG ALS datasets, underscoring the robustness and generalizability of both models across diverse cohorts.

Despite its slightly lower discrimination, the RP model offers unique strengths. Unlike XGBoost's binary classification (death within 6 or 12 months), RP provides continuous survival probabilities and full survival curves, which are more informative when precise estimates of survival duration are needed. Additionally, RP's coefficients are interpretable as log‐hazard ratios, which show how each feature increases or decreases the risk of death, making it more explainable.

XGBoost, on the other hand, balances strong predictive performance with practical advantages. It handles missing data natively, performs well across heterogeneous datasets, and relies only on routinely collected clinical inputs. Though the gains in AUC over RP were modest (Figures 1 and 2), even small improvements may be clinically meaningful in high‐stakes settings like end‐of‐life prognostication. Nevertheless, such marginal gains must be weighed against increased complexity and implementation demands.

Ultimately, the choice between RP and XGBoost depends on the intended use. Our results provide a comparative framework to guide model selection based on clinical context, balancing interpretability, usability, and performance rather than prescribing a single best approach.

### 4.3 Feature Importance

Consistent with prior studies, we identified increased age [37], bulbar onset [38], higher rate of decline in ALSFRS‐R scores [39] and low BMI [40] as factors associated with earlier mortality. While the prognostic value of serum albumin is less frequently highlighted, studies have shown that lower albumin at diagnosis correlates with worse outcomes [41].

Our XGBoost analysis of the PRO‐ACT database consistently identified albumin as an important feature for mortality prediction, and subsequent correlation analysis demonstrated a significant linear relationship between albumin levels and survival duration. Since albumin reflects both nutritional status and inflammation [41, 42], and can be increased by managing inflammation and providing nutritional support [43, 44], these findings support monitoring albumin alongside established clinical measures. Nevertheless, albumin's predictive power is strongest within a multivariable context. In our feature‐reduction analysis (Figure S4), model accuracy declined sharply when fewer than seven top‐ranked features were included. Other features must therefore be considered alongside albumin to achieve robust mortality predictions.

Additionally, our study identified laboratory markers such as bicarbonate and chloride (markers of respiratory compromise) [45], phosphorus (sign of energy imbalance) [46], absolute basophil and eosinophil counts (reflect systemic inflammation) [47], and protein (nutritional status) as potential correlates of mortality. These findings provide insights into ALS prognosis and highlight the importance of integrating clinical and laboratory data for more precise and timely mortality predictions. While these biomarkers do not directly measure motor neuron loss, they may help identify patients with better or worse prognoses earlier, offering potential avenues to understand disease mechanisms and develop interventions before irreversible motor neuron degeneration occurs.

### 4.4 Limitations and Future Directions

In this study, we focused on predicting 6‐and 12‐month mortality due to their importance for clinical trials and patient care decisions. The prediction period was also limited by the PRO‐ACT database, where over half of the patients died within 12 months and 20% died within 6 months after any clinical visit. We were hence unable to reliably assess very short or very long survival times. While AUC remained high on the SG ALS dataset, which has patients with much longer survival times, the sensitivity, PPV, and AUPRC decreased due to the low death prevalence. Model performance may decline in patients whose functional status or survival trajectories differ significantly from those in our training data (Figure S1). Future models should include broader survival profiles to improve generalizability.

Although we validated our models on both a North American trial cohort and an Asian clinical population, performance in other geographic or clinical contexts remains unknown. Future studies should continue to evaluate the models across different geographical and clinical settings, potentially retraining on local datasets to improve demographic and clinical fit.

Missing data posed another limitation. We excluded visits with > 50% missing labs to ensure quality, but this may introduce bias if missingness correlates with disease severity. Also, our primary imputation approach used linear interpolation, which relies on future data and is not feasible in real‐time clinical use. However, we showed that performance remained robust when using KNN‐only imputation limited to information available at the time of prediction. In future work, we aim to explore models that treat missingness as a predictive signal and include broader clinical populations.

Some clinically important variables, such as frontotemporal dementia status and genetic testing, were unavailable across PRO‐ACT and thus excluded. While these may enhance predictive performance, they are not routinely collected in all settings and may be especially limited in low‐resource environments. Our models also lacked direct data on noninvasive ventilatory (NIV) support (e.g., BiPAP), a key indicator of respiratory muscle weakness and later ALS stage. Although our models performed well without it, relying on surrogate markers like FVC, respiratory rate, bicarbonate, ALSFRS‐R, and albumin, direct inclusion of ventilatory support data would likely improve clinical utility. Future models incorporating richer, prospectively collected clinical, cognitive, genetic, and NIV data may improve prognostic accuracy.

## 5 Conclusion

ML is a powerful tool for studying and managing complex neuromuscular diseases such as ALS, which is known to be biologically and clinically heterogenous [48]. We developed a set of models that can be used to predict mortality in ALS patients, enabling healthcare professionals to make care recommendations that are best tailored to a patient's expected clinical course. We also identified a set of features that are important for mortality prediction. Our approach signifies a step towards harnessing the power of predictive modeling to guide healthcare providers, their patients, and caregivers as they consider goals of care, advance directives, and end‐of‐life decisions. Such a tool would be invaluable not only in enhancing the quality of end‐of‐life care but also in boosting the efficacy of clinical trial design and execution.

## Author Contributions

**Ling Guo:** methodology, formal analysis, writing – original draft, writing – review and editing. **Ian Qian Xu:** methodology, formal analysis, writing – original draft, writing – review and editing. **Sonakshi Nag:** writing – original draft, writing – review and editing, formal analysis. **Jing Xu:** methodology, writing – review and editing. **Josiah Chai:** writing – review and editing, data curation. **Zachary Simmons:** writing – original draft, writing – review and editing, formal analysis. **Savitha Ramasamy:** conceptualization, supervision, writing – original draft, writing – review and editing, formal analysis, methodology. **Crystal Jing Jing Yeo:** conceptualization, supervision, writing – original draft, writing – review and editing, formal analysis, funding acquisition, methodology.

## Ethics Statement

We confirm that we have read the journal's position on issues involved in ethical publication and affirm that this report is consistent with those guidelines.

## Conflicts of Interest

Dr. Zachary Simmons, one of the co‐authors of this manuscript, currently serves as the Editor‐in‐Chief of *Muscle & Nerve*. To avoid any conflicts of interest, editorial handling and peer review of this manuscript were managed independently by another member of the editorial board.

## Supporting information


**Data S1.** Supporting Information.

## Data Availability

The PRO‐ACT data is publicly available at https://ncri1.partners.org/proact. The Celecoxib trial dataset is provided through DTUA2021A009305 with Massachusetts General Hospital/Harvard Medical School and the NEALS consortium. The SG ALS dataset and analysis code are available from the corresponding authors upon reasonable request.

## References

[1] A. Chiò , G. Logroscino , O. Hardiman , et al., “Prognostic Factors in ALS: A Critical Review,” Amyotrophic Lateral Sclerosis 10 (2009): 310–323. 19922118 10.3109/17482960802566824 PMC3515205

[2] E. Longinetti and F. Fang , “Epidemiology of Amyotrophic Lateral Sclerosis: An Update of Recent Literature,” Current Opinion in Neurology 32 (2019): 771–776. 31361627 10.1097/WCO.0000000000000730 PMC6735526

[3] B. Sever , H. Ciftci , H. Demirci , et al., “Comprehensive Research on Past and Future Therapeutic Strategies Devoted to Treatment of Amyotrophic Lateral Sclerosis,” International Journal of Molecular Sciences 23 (2022): 2400. 35269543 10.3390/ijms23052400 PMC8910198

[4] F. Cheng , F. Wang , J. Tang , et al., “Artificial Intelligence and Open Science in Discovery of Disease‐Modifying Medicines for Alzheimer's Disease,” Cell Reports Medicine 5, no. 2 (2024): 101379, 10.1016/j.xcrm.2023.101379. 38382465 PMC10897520

[5] C. Su , Y. Hou , J. Xu , et al., “Identification of Parkinson's Disease PACE Subtypes and Repurposing Treatments Through Integrative Analyses of Multimodal Data,” npj Digital Medicine 7 (2024), 10.1038/s41746-024-01175-9. PMC11233682 38982243

[6] C. N. Fournier , “Considerations for Amyotrophic Lateral Sclerosis (ALS) Clinical Trial Design,” Neurotherapeutics 19 (2022): 1180–1192. 35819713 10.1007/s13311-022-01271-2 PMC9275386

[7] Y. Huang , X. Wu , and R. H. M. Chan , “Stratification and Survival Prediction for Amyotrophic Lateral Sclerosis Patients,” in BHI‐BSN 2022‐IEEE‐EMBS International Conference on Biomedical and Health Informatics and IEEE‐EMBS International Conference on Wearable and Implantable Body Sensor Networks, Symposium Proceedings. Institute of Electrical and Electronics Engineers Inc (IEEE, 2022).

[8] M. Elamin , P. Bede , A. Montuschi , N. Pender , A. Chio , and O. Hardiman , “Predicting Prognosis in Amyotrophic Lateral Sclerosis: A Simple Algorithm,” Journal of Neurology 262 (2015): 1447–1454. 25860344 10.1007/s00415-015-7731-6 PMC4469087

[9] A. L. Kjældgaard , K. Pilely , K. S. Olsen , et al., “Prediction of Survival in Amyotrophic Lateral Sclerosis: A Nationwide, Danish Cohort Study,” BMC Neurology 21 (2021): 1–8. 33865343 10.1186/s12883-021-02187-8 PMC8052712

[10] C. Lunetta , A. Lizio , M. G. Melazzini , E. Maestri , and V. A. Sansone , “Amyotrophic Lateral Sclerosis Survival Score (ALS‐SS): A Simple Scoring System for Early Prediction of Patient Survival,” Amyotrophic Lateral Sclerosis and Frontotemporal Degeneration 17 (2016): 93–100. 10.3109/21678421.2015.1083585 26470943

[11] H.‐J. Westeneng , T. P. A. Debray , A. E. Visser , et al., “Prognosis for Patients With Amyotrophic Lateral Sclerosis: Development and Validation of a Personalised Prediction Model,” Lancet Neurology 17 (2018): 423–433. 29598923 10.1016/S1474-4422(18)30089-9

[12] F. Papaiz , M. E. Dourado, Jr. , R. A. Valentim , A. H. de Morais , and J. P. Arrais , “Machine Learning Solutions Applied to Amyotrophic Lateral Sclerosis Prognosis: A Review,” Frontiers in Computer Science 4 (2022): 869140.

[13] M. L. Ong , P. F. Tan , and J. D. Holbrook , “Predicting Functional Decline and Survival in Amyotrophic Lateral Sclerosis,” PLoS One 12 (2017): e0174925. 28406915 10.1371/journal.pone.0174925 PMC5390993

[14] S. R. Pfohl , R. B. Kim , G. S. Coan , and C. S. Mitchell , “Unraveling the Complexity of Amyotrophic Lateral Sclerosis Survival Prediction,” Frontiers in Neuroinformatics 12 (2018): 346832. 10.3389/fninf.2018.00036 PMC6010549 29962944

[15] C. Schuster , O. Hardiman , and P. Bede , “Survival Prediction in Amyotrophic Lateral Sclerosis Based on MRI Measures and Clinical Characteristics,” BMC Neurology 17 (2017): 1–10. 28412941 10.1186/s12883-017-0854-x PMC5393027

[16] H. K. van der Burgh , R. Schmidt , H. J. Westeneng , M. A. de Reus , L. H. van den Berg , and M. P. van den Heuvel , “Deep Learning Predictions of Survival Based on MRI in Amyotrophic Lateral Sclerosis,” NeuroImage: Clinical 13 (2017): 361–369. 28070484 10.1016/j.nicl.2016.10.008 PMC5219634

[17] M. A. Din Abdul Jabbar , L. Guo , Y. Guo , et al., “Describing and Characterising Variability in ALS Disease Progression,” Amyotrophic Lateral Sclerosis and Frontotemporal Degeneration 25 (2024): 34–45. 37794802 10.1080/21678421.2023.2260838

[18] P. H. Gordon , B. Cheng , F. Salachas , et al., “Progression in ALS Is Not Linear but Is Curvilinear,” Journal of Neurology 257 (2010): 1713–1717. 20532545 10.1007/s00415-010-5609-1

[19] M. Yu , J. Xu , R. Dutta , B. Trapp , A. A. Pieper , and F. Cheng , “Network Medicine Informed Multiomics Integration Identifies Drug Targets and Repurposable Medicines for Amyotrophic Lateral Sclerosis,” npj Systems Biology and Applications 10 (2024): 128. 39500920 10.1038/s41540-024-00449-y PMC11538253

[20] Q. H. Sun , Y. R. Li , W. J. Lan , F. Yang , F. Cui , and X. S. Huang , “Prognostic Value of Time to Generalization in 71 Chinese Patients With Sporadic Amyotrophic Lateral Sclerosis,” Chinese Medical Journal 132 (2019): 1023–1027. 31033570 10.1097/CM9.0000000000000200 PMC6595875

[21] L. Xu , B. He , Y. Zhang , et al., “Prognostic Models for Amyotrophic Lateral Sclerosis: A Systematic Review,” Journal of Neurology 268 (2021): 3361–3370. 33694050 10.1007/s00415-021-10508-7

[22] Q. Wei , X. Chen , Z. Zheng , et al., “The Predictors of Survival in Chinese Amyotrophic Lateral Sclerosis Patients,” Amyotrophic Lateral Sclerosis and Frontotemporal Degeneration 16 (2015): 237–244. 25581512 10.3109/21678421.2014.993650

[23] M. E. Cudkowicz , J. M. Shefner , D. A. Schoenfeld , et al., “Trial of Celecoxib in Amyotrophic Lateral Sclerosis,” Annals of Neurology 60 (2006): 22–31. 16802291 10.1002/ana.20903

[24] I. Q. Xu , L. Guo , J. Xu , et al., “Predictive Analysis of Amyotrophic Lateral Sclerosis Progression and Mortality in a Clinic Cohort From Singapore,” Muscle & Nerve 72, no. 1 (2025): 71–81, 10.1002/mus.28416. 40265300 PMC12138491

[25] T. Chen and C. Guestrin , “XGBoost: A Scalable Tree Boosting System,” in Proceedings of the 22nd Acm Sigkdd International Conference on Knowledge Discovery and Data Mining (ACM, 2016), 785–794.

[26] V. Grollemund , P. F. Pradat , G. Querin , et al., “Machine Learning in Amyotrophic Lateral Sclerosis: Achievements, Pitfalls, and Future Directions,” Frontiers in Neuroscience 13 (2019): 135. 30872992 10.3389/fnins.2019.00135 PMC6403867

[27] M. A. Din Abdul Jabbar , L. Guo , S. Nag , et al., “Predicting Amyotrophic Lateral Sclerosis (ALS) Progression With Machine Learning,” Amyotrophic Lateral Sclerosis and Frontotemporal Degeneration 25 (2024): 242–255. 38052485 10.1080/21678421.2023.2285443

[28] C. Pancotti , G. Birolo , T. Sanavia , C. Rollo , and P. Fariselli , “Multi‐Event Survival Prediction for Amyotrophic Lateral Sclerosis,” in CEUR Workshop Proceedings, Vol. 3180, ed. G. Faggioli , N. Ferro , A. Hanbury , and M. Potthast (CEUR Workshop Proceedings (CEUR‐WS.org), 2022), 1269–1276.

[29] N. Atassi , J. Berry , A. Shui , et al., “The PRO‐ACT Database: Design, Initial Analyses, and Predictive Features,” Neurology 83 (2014): 1719–1725. 25298304 10.1212/WNL.0000000000000951 PMC4239834

[30] C. Jackson , “Flexsurv: A Platform for Parametric Survival Modeling in R,” Journal of Statistical Software 70 (2016): i08. 29593450 10.18637/jss.v070.i08 PMC5868723

[31] Y. Xie , B. Jiang , E. Gong , et al., “Use of Gradient Boosting Machine Learning to Predict Patient Outcome in Acute Ischemic Stroke on the Basis of Imaging, Demographic, and Clinical Information,” American Journal of Roentgenology 212 (2019): 44–51. 30354266 10.2214/AJR.18.20260

[32] M. Lee , N. Y. Yeo , H. J. Ahn , et al., “Prediction of Post‐Stroke Cognitive Impairment After Acute Ischemic Stroke Using Machine Learning,” Alzheimer's Research & Therapy 15 (2023): 1–10. 10.1186/s13195-023-01289-4 PMC10468853 37653560

[33] D. Ramamoorthy , K. Severson , S. Ghosh , K. Sachs , and Answer ALS , “Identifying Patterns in Amyotrophic Lateral Sclerosis Progression From Sparse Longitudinal Data,” Nature Computational Science 2 (2022): 605–616. 38177466 10.1038/s43588-022-00299-w PMC10766562

[34] N. J. Thakore , B. R. Lapin , and E. P. Pioro , “Trajectories of Impairment in Amyotrophic Lateral Sclerosis: Insights From the Pooled Resource Open‐Access ALS Clinical Trials Cohort,” Muscle & Nerve 57, no. 6 (2018): 937–945, 10.1002/mus.26042. 29244213

[35] R. G. Huber , S. Pandey , D. Chhangani , D. E. Rincon‐Limas , N. P. Staff , and C. J. J. Yeo , “Identification of Potential Pathways and Biomarkers Linked to Progression in ALS,” Annals of Clinical and Translational Neurology 10 (2023): 150–165. 36533811 10.1002/acn3.51697 PMC9930436

[36] B. Huang , X. Geng , Z. Yu , C. Zhang , and Z. Chen , “Dynamic Effects of Prognostic Factors and Individual Survival Prediction for Amyotrophic Lateral Sclerosis Disease,” Annals of Clinical and Translational Neurology 10 (2023): 892–903. 37014017 10.1002/acn3.51771 PMC10270250

[37] A. M. Chancellor , J. M. Slattery , H. Fraser , R. J. Swingler , S. M. Holloway , and C. P. Warlow , “The Prognosis of Adult‐Onset Motor Neuron Disease: A Prospective Study Based on the Scottish Motor Neuron Disease Register,” Journal of Neurology 240 (1993): 339–346. 8336173 10.1007/BF00839964

[38] E. S. Louwerse , C. E. Visser , P. M. M. Bossuyt , and G. J. Weverling , “Amyotrophic Lateral Sclerosis: Mortality Risk During the Course of the Disease and Prognostic Factors,” Journal of the Neurological Sciences 152 (1997): s10–s17. 9419048 10.1016/s0022-510x(97)00238-4

[39] A. Chiò , G. Mora , M. Leone , et al., “Early Symptom Progression Rate Is Related to ALS Outcome: A Prospective Population‐Based Study,” Neurology 59 (2002): 99–103. 12105314 10.1212/wnl.59.1.99

[40] S. Paganoni , J. Deng , M. Jaffa , M. E. Cudkowicz , and A. M. Wills , “Body Mass Index, Not Dyslipidemia, is an Independent Predictor of Survival in Amyotrophic Lateral Sclerosis,” Muscle Nerve 44 (2011): 20–24. 21607987 10.1002/mus.22114 PMC4441750

[41] A. Chiò , A. Calvo , G. Bovio , et al., “Amyotrophic Lateral Sclerosis Outcome Measures and the Role of Albumin and Creatinine: A Population‐Based Study,” JAMA Neurology 71 (2014): 1134–1142. 25048026 10.1001/jamaneurol.2014.1129

[42] B. Chełstowska and M. Kuźma‐Kozakiewicz , “Biochemical Parameters in Determination of Nutritional Status in Amyotrophic Lateral Sclerosis,” Neurological Sciences 41 (2020): 1115–1124. 31897946 10.1007/s10072-019-04201-x

[43] B. R. Don and G. Kaysen , “Poor Nutritional Status and Inflammation: Serum Albumin: Relationship to Inflammation and Nutrition,” Seminars in Dialysis 17 (2004): 432–437. 15660573 10.1111/j.0894-0959.2004.17603.x

[44] P. B. Soeters , R. R. Wolfe , and A. Shenkin , “Hypoalbuminemia: Pathogenesis and Clinical Significance,” Journal of Parenteral and Enteral Nutrition 43 (2019): 181–193. 30288759 10.1002/jpen.1451 PMC7379941

[45] J. Foucher , T. Wellander , A. Lovik , et al., “Venous Bicarbonate as a Prognostic Biomarker and Proposed Proxy for Vital Capacity to be Used as an Eligibility Criterion in Amyotrophic Lateral Sclerosis Clinical Trials,” Brain and Behavior: A Cognitive Neuroscience Perspective 15 (2025): e70570. 10.1002/brb3.70570 PMC12086301 40383997

[46] R. Vasta , E. Koumantakis , A. Canosa , et al., “Phosphatemia Is an Independent Prognostic Factor in Amyotrophic Lateral Sclerosis,” Annals of Neurology 98, no. 2 (2025): 286–293, 10.1002/ana.27252. 40285624 PMC12278033

[47] M. Grassano , U. Manera , F. De Marchi , et al., “The Role of Peripheral Immunity in ALS: A Population‐Based Study,” Annals of Clinical and Translational Neurology 10 (2023): 1623–1632. 37482930 10.1002/acn3.51853 PMC10502618

[48] C. J. J. Yeo , S. Ramasamy , F. J. Leong , S. Nag , and Z. Simmons , “A Neuromuscular Clinician's Primer on Machine Learning,” Journal of Neuromuscular Diseases (2025): 22143602251329240, 10.1177/22143602251329240. 40165764 PMC13141858

