# Supplementary material for: Predicting Amyotrophic Lateral Sclerosis Mortality With Machine Learning in Diverse Patient Databases
Source: Muscle Nerve. 2025 Jul 28;72(4):653–61. doi: 10.1002/mus.28487 (PMC12435129; doi:10.1002/mus.28487)
Supplement: Supplementary file 1 — Data S1. Supporting Information. [file MUS-72-653-s001.docx]

**Supplementary information**

**Supplementary figures**


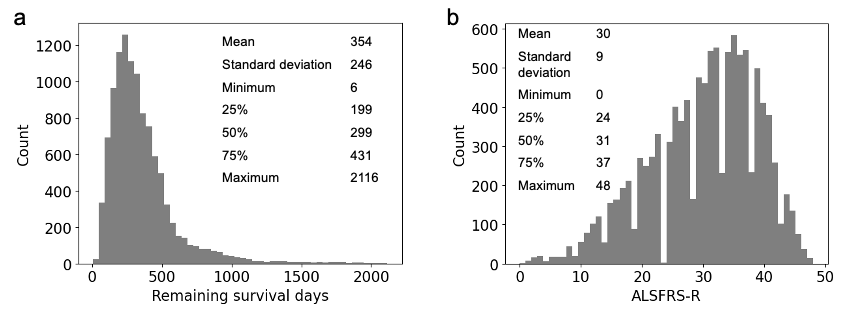


**Supp. Fig. 1** Histograms of the (a) remaining survival days and (b) ALSFRS-R scores of all visits of all patients included in the study from the PRO-ACT database. The table in the plot shows the mean, standard deviation, minimum, maximum, and 25^th^, 50^th^ and 75^th^ percentiles of the distribution.

**
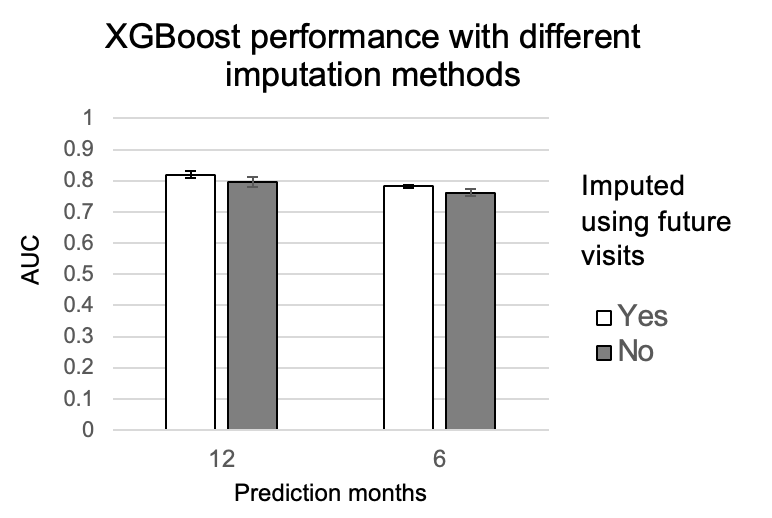
**

**Supp. Fig. 2** Area Under the Receiver operating characteristics curve (AUC) for 12 and 6 months mortality prediction using different missing data imputation methods. White bars show performance for data imputed using linear interpolation, which may include information from future visits, and k-Nearest Neighbors (KNN). Shaded bars show performance for data imputed using KNN only, which does not require information from future visits of the patient.

*
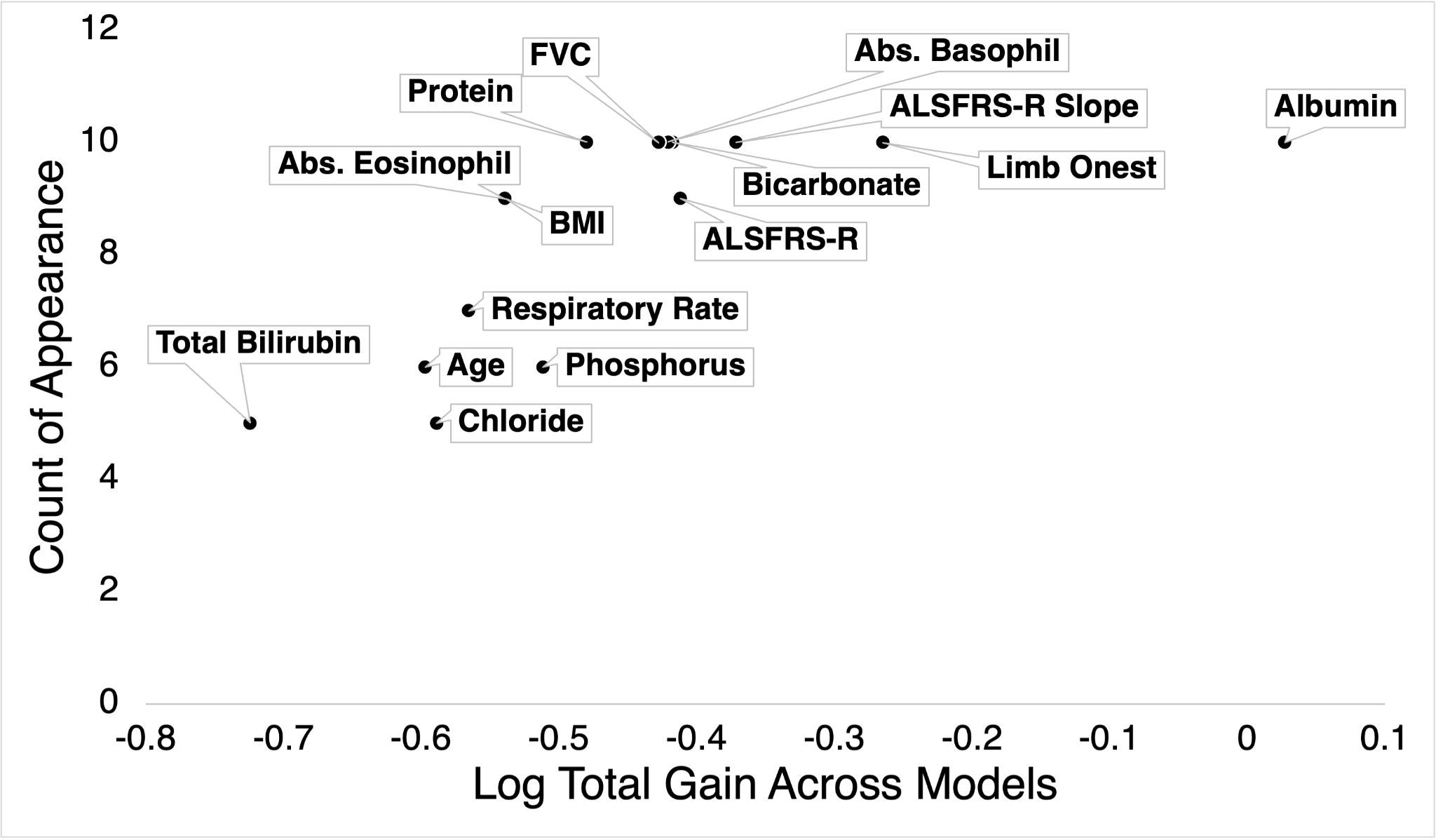
*

**Supp. Figure 3** Top 15 features important for 12 and 6-month mortality prediction for XGBoost models


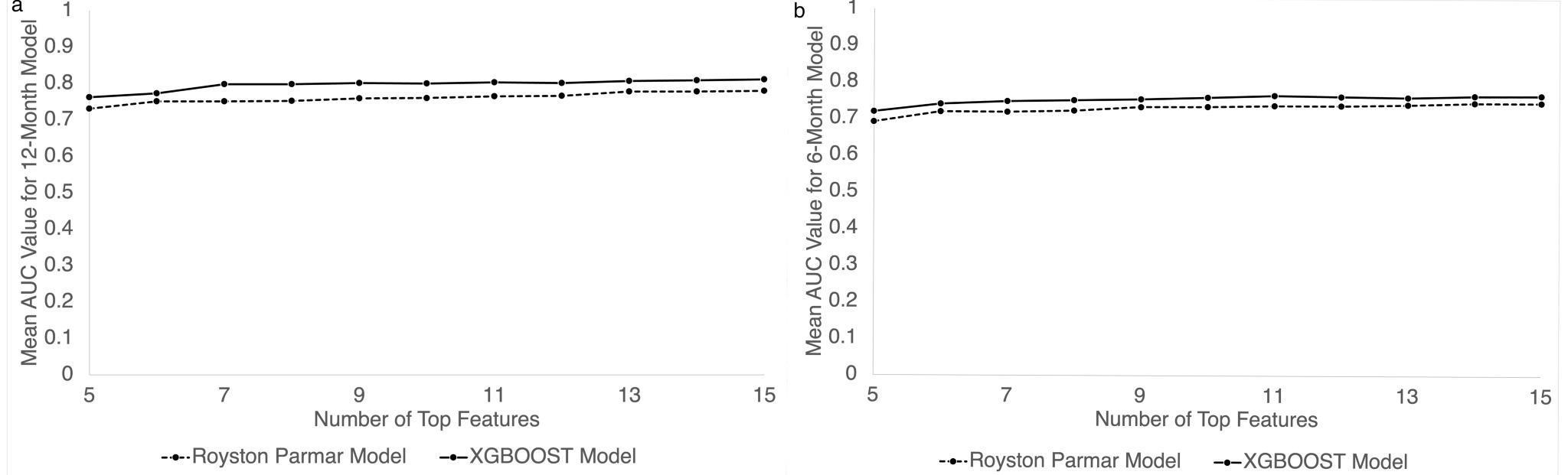


**Supp. Figure 4** ALS mortality prediction AUC comparison between Royston Parmar model and XGBoost model with different number of predictors on the PRO-ACT database

**Supplementary tables**

**Supp. Table 1** All Features Used in PRO-ACT Database

| **Category** | **Parameter** | **Units** | **Percentage missing in training and test data** | **Percentage Missing in PRO-ACT Database** |
| --- | --- | --- | --- | --- |
| Demographic | Sex^ |  | 0.00% | 0.00% |
|  | Age^ | years | 0.00% | 28.20% |
|  | Site (bulbar/limb/both/other)^ |  | 0.00% | 12.40% |
| Clinical | Days since Onset of Symptoms | days | 0% | 35.90% |
|  | Days since diagnosis | days | 39.70% | 63.10% |
|  | Riluzole Use |  | 22.40% | 17.80% |
|  | BMI | kg/m^2 | 28.50% | 23.80% |
|  | FVC (as % of Predicted for Normal Individual) | proportion | 33.60% | 6.70% |
|  | Diastolic Blood Pressure | mmHg | 17.70% | 21.00% |
|  | Systolic Blood Pressure | mmHg | 17.70% | 21.00% |
|  | Pulse | bpm | 17.50% | 21.00% |
|  | Respiratory Rate | breaths/min | 27.10% | 45.40% |
|  | ALSFRS-R |  | 0.00% | 36.1% |
| Laboratory | ALT | U/L | 21.20% | 5.20% |
|  | AST | U/L | 21.40% | 9.50% |
|  | Creatinine | μmol/L | 21.10% | 21.00% |
|  | CK | U/L | 32.10% | 39.80% |
|  | Total Cholesterol* | mmol/L | 43.10% | 52.10% |
|  | Triglycerides* | mmol/L | 46.90% | 55.10% |
|  | HbA1c* | % | 62.00% | 71.40% |
|  | Glucose | mmol/L | 21.20% | 25.00% |
|  | Phosphorus | mmol/L | 28.80% | 44.30% |
|  | Sodium | mmol/L | 21.10% | 22.40% |
|  | Potassium | mmol/L | 21.30% | 22.60% |
|  | Chloride | mmol/L | 24.30% | 24.60% |
|  | Calcium | mmol/L | 22.00% | 32.50% |
|  | Bicarbonate | mmol/L | 30.70% | 30.20% |
|  | BUN | mmol/L | 21.10% | 24.90% |
|  | Uric Acid* | μmol/L | 72.80% | 70.60% |
|  | Alkaline Phosphatase | U/L | 0.10% | 28.80% |
|  | GGT | U/L | 43.70% | 39.20% |
|  | Bilirubin | μmol/L | 21.10% | 37.00% |
|  | Protein | g/L | 29.30% | 39.60% |
|  | Albumin | g/L | 22.00% | 34.50% |
|  | Hb | g/L | 1.60% | 17.60% |
|  | Haematocrit | % | 22.00% | 17.90% |
|  | RBC | 10E9/L | 28.10% | 18.40% |
|  | WBC | 10E9/L | 28.10% | 18.40% |
|  | Platelets | 10E9/L | 29.10% | 25.30% |
|  | Absolute Eosinophil Count | 10E9/L | 30.80% | 27.50% |
|  | Eosinophils Percentage | % | 41.30% | 49.00% |
|  | Absolute Lymphocyte Count | 10E9/L | 42.60% | 35.10% |
|  | Lymphocyte Percentage | % | 29.50% | 41.30% |
|  | Absolute Basophil Count | 10E9/L | 36.40% | 35.20% |
|  | Basophil Percentage | % | 29.60% | 41.30% |
|  | Absolute Monocyte Count | 10E9/L | 42.60% | 35.10% |
|  | Monocyte Percentage | % | 29.50% | 41.30% |
|  | Absolute Neutrophil Count | 10E9/L | 42.60% | 35.10% |

**Supp. Table 2** All features used in Celecoxib trial database

| **Category** | **Parameter** | **Units** | **Percentage Missing** |
| --- | --- | --- | --- |
| Demographic | Sex^ |  | 0.0% |
|  | Age^ | years | 0.0% |
|  | Site (bulbar/limb/both/other)^ |  | 0.0% |
| Clinical | Days since Onset of Symptoms | days | 0.0% |
|  | Days since diagnosis | days | 0.0% |
|  | Riluzole Use |  | 0.0% |
|  | BMI | kg/m^2 | 26.8% |
|  | FVC (as % of Predicted for Normal Individual)^ | proportion | 26.6% |
|  | Diastolic Blood Pressure | mmHg | 25.2% |
|  | Systolic Blood Pressure | mmHg | 25.2% |
|  | Pulse | bpm | 25.2% |
|  | Respiratory Rate | breaths/min | 25.6% |
|  | ALSFRS-R |  | 0.0% |
| Laboratory | ALT | U/L | 16.8% |
|  | AST | U/L | 16.8% |
|  | Creatinine | μmol/L | 16.6% |
|  | Glucose | mmol/L | 99.2% |
|  | Sodium | mmol/L | 16.5% |
|  | Potassium | mmol/L | 16.6% |
|  | Chloride | mmol/L | 16.5% |
|  | Bicarbonate | mmol/L | 16.6% |
|  | BUN | mmol/L | 16.7% |
|  | Bilirubin | μmol/L | 17.3% |
|  | Hb | g/L | 16.8% |
|  | Haematocrit | % | 16.8% |
|  | RBC | 10E9/L | 16.8% |
|  | WBC | 10E9/L | 16.8% |

**Supp. Table 3** All features used in Singapore database

| **Category** | **Parameter** | **Units** | **Percentage Missing** |
| --- | --- | --- | --- |
| Demographic | Sex |  | 0 |
|  | Age | years | 0 |
|  | Site (bulbar/limb) |  | 0 |
| Clinical | Days since Onset of Symptoms | days | 0 |
|  | Days since diagnosis | days | 0 |
|  | Riluzole Use |  | 0 |
|  | Height | m | 0 |
|  | Weight | kg | 92.9 |
|  | FVC (as % of Predicted for Normal Individual) | proportion | 87.9 |
|  | Pulse | bpm | 98.4 |
|  | ALSFRS-R |  | 73.8 |
| Laboratory | WBC | 10E9/L | 47.0 |
|  | RBC | 10E9/L | 47.1 |
|  | Platelets | 10E9/L | 47.0 |
|  | AbsNeutroCount | 10E9/L | 48.2 |
|  | AbsEosinophil | 10E9/L | 47.6 |
|  | AbsBasophil | 10E9/L | 47.6 |
|  | Creatinine | μmol/L | 43.8 |
|  | Albumin | g/L | 47.9 |
|  | BilirubinTotal | μmol/L | 70.1 |
|  | AST | U/L | 63.2 |
|  | ALT | U/L | 56.0 |
|  | Alkaline Phosphatase | U/L | 58.8 |
|  | CK | U/L | 76.2 |
|  | Bicarbonate | mmol/L | 86.4 |
|  | Chloride | mmol/L | 97.3 |
|  | Phosphorus | mmol/L | 92.1 |
|  | HbA1c | % | 98.6 |
|  | Protein | g/L | 98.3 |

**​​Supp. Table 4** Comparison of the features in the three databases

|  | **PRO-ACT (Mean ± SD)** | **Singapore** | **Celecoxib** | **p-value (PRO-ACT vs. SG)** | **p-value (PRO-ACT vs. Celecoxib)** |
| --- | --- | --- | --- | --- | --- |
| **Age** | 55.53 ± 11.42 | 57.64 ± 10.36 | 55.02 ± 12.14 | <0.001 | 0.024 |
| **BMI** | 25.13 ± 4.58 | 20.49 ± 3.97 | 26.5 ± 4.49 | <0.001 | <0.001 |
| **Pulse** | 78.68 ± 12.47 | 84 ± 17.85 | 76.93 ± 12.87 | 0.051 | <0.001 |
| **Respiratory Rate** | 18.05 ± 3.74 |  | 18.21 ± 3.48 |  | 0.058 |
| **Diastolic BP** | 80.76 ± 10.34 |  | 79.82 ± 10.61 |  | <0.001 |
| **Systolic BP** | 128.33 ± 16.39 |  | 129.83 ± 16.17 |  | <0.001 |
| **FVC** | 0.72 ± 0.24 | 0.68 ± 0.22 | 0.76 ± 0.22 | 0.049 | <0.001 |
| **ALT** | 34.11 ± 23.87 | 35.59 ± 23.3 | 34.62 ± 17.7 | <0.001 | 0.324 |
| **AST** | 29.53 ± 14.14 | 34.91 ± 15.82 | 31.45 ± 11.7 | <0.001 | <0.001 |
| **Uric Acid** | 294.2 ± 80.85 |  |  |  |  |
| **BUN** | 5.59 ± 1.7 |  | 5.8 ± 1.81 |  | <0.001 |
| **Albumin** | 43.23 ± 3.54 | 40.09 ± 5.27 |  | <0.001 |  |
| **Abs. Neutrophil** | 4.58 ± 1.62 | 5.17 ± 3.14 |  | <0.001 |  |
| **Protein** | 71.86 ± 4.74 | 72.05 ± 5.58 |  | 0.856 |  |
| **CK** | 286.84 ± 280.29 | 261.08 ± 222.41 |  | 0.105 |  |
| **Cholesterol** | 5.78 ± 1.14 |  |  |  |  |
| **Triglycerides** | 2.01 ± 1.35 |  |  |  |  |
| **HbA1c** | 5.45 ± 0.74 | 5.69 ± 0.76 |  | 0.105 |  |
| **Hb** | 144.32 ± 13 |  | 144.4 ± 19.51 |  | 0.783 |
| **Hematocrit** | 43.43 ± 3.97 |  | 42.32 ± 4.2 |  | <0.001 |
| **WBC** | 6.82 ± 2.19 | 7.63 ± 3.36 | 7.02 ± 2.09 | <0.001 | <0.001 |
| **RBC** | 4711.89 ± 501.42 | 4384.22 ± 502.01 | 4667.42 ± 453.81 | <0.001 | <0.001 |
| **Creatinine** | 65.6 ± 20. | 47.29 ± 20.56 | 71.31 ± 23.74 | <0.001 | <0.001 |
| **Sodium** | 140.23 ± 2.73 |  | 139.85 ± 2.85 |  | <0.001 |
| **Potassium** | 4.19 ± 0.34 |  | 4.2 ± 0.38 |  | 0.173 |
| **Chloride** | 102.47 ± 3.5 | 101.31 ± 3.38 | 102.98 ± 3.15 | 0.050 | <0.001 |
| **Glucose** | 5.64 ± 1.63 |  | 5.37 ± 2.38 |  | 0.449 |
| **Platelets** | 249.45 ± 64.59 | 274.13 ± 103.43 |  | <0.001 |  |
| **Abs. Eosinophil** | 0.5 ± 1.22 | 0.14 ± 0.19 |  | <0.001 |  |
| **Alkaline Phosphatase** | 78.12 ± 28.38 | 81.29 ± 50.15 |  | 0.011 |  |
| **Bicarbonate** | 26.04 ± 3.43 | 27.55 ± 4.97 | 26.23 ± 3.14 | <0.001 | 0.012 |
| **Calcium** | 2.37 ± 0.12 |  |  |  |  |
| **Abs. Lymphocyte** | 1.73 ± 0.58 |  |  |  |  |
| **Abs. Monocyte** | 0.43 ± 0.18 |  |  |  |  |
| **Abs. Basophil** | 0.04 ± 0.03 | 0.04 ± 0.08 |  | <0.001 |  |
| **Total Bilirubin** | 10.36 ± 5.89 | 15.9 ± 7.72 | 11.21 ± 6.16 | <0.001 | <0.001 |
| **GGT** | 36.4 ± 42.97 |  |  |  |  |
| **Lymphocytes Perc** | 25.76 ± 7.62 |  |  |  |  |
| **Monocytes Perc** | 6.22 ± 2.35 |  |  |  |  |
| **Basophils Perc** | 0.62 ± 0.45 |  |  |  |  |
| **Phosphorus** | 1.21 ± 0.17 | 1.04 ± 0.24 |  | <0.001 |  |
| **Eosinophils Perc** | 2.31 ± 1.66 |  |  |  |  |
| **ALSFRS-R** | 31.62 ± 9.34 | 32.04 ± 9.66 | 35.14 ± 7.73 | 0.399 | <0.001 |
| **Disease Duration** | 23.00 ± 17.00 | 62.12 ± 41.68 | 32.38 ± 12.20 | <0.001 | <0.001 |
| **Diagnostic Delay** | 11.60 ± 9.20 | 14.44 ± 14.35 | 12.96 ± 10.8 | <0.001 | <0.001 |

**Supp. Table 5** Mortality prediction using the subset of ENCALS features available in PRO-ACT: FVC, diagnostic delay, age of onset, site of onset, ALSFRS-R slope

| Model | Royston-Parmar | XGBoost | Royston-Parmar | XGBoost |
| --- | --- | --- | --- | --- |
| Prediction months | 12 | | 6 | |
| Training size | 8458 ± 109 | | 8782 ± 116 | |
| Test size | 2113 ± 109 | | 2194 ± 116 | |
| % died | 61.6 ± 1.9 | | 20.3 ± 1.1 | |
| AUC | 0.715 ± 0.023 | 0.743 ± 0.010 | 0.677 ± 0.016 | 0.717 ± 0.017 |
| AUPRC | 0.484 ± 0.024 | 0.806 ± 0.022 | 0.142 ± 0.01 | 0.397 ± 0.026 |
| Accuracy | 0.678 ± 0.015 | 0.690 ± 0.007 | 0.596 ± 0.058 | 0.655 ± 0.039 |
| Sensitivity | 74.1 ± 4.4 | 73.7 ± 2.6 | 64.6 ± 9.8 | 64.9 ± 6.8 |
| Specificity | 57.6 ± 5.5 | 61.5 ± 3.0 | 58.2 ± 9.7 | 65.7 ± 6.6 |
| F1 | 0.739 ± 0.023 | 0.745 ± 0.013 | 0.393 ± 0.024 | 0.433 ± 0.015 |
| PPV | 73.8 ± 1.7 | 75.4 ± 1.6 | 28.5 ± 1.8 | 32.7 ± 2.4 |
| NPV | 58.2 ± 2.2 | 59.3 ± 1.9 | 86.8 ± 1.5 | 88.1 ± 1.2 |

**Supp. Table 6** Mortality prediction using all PROACT features

| Model | Royston-Parmar | XGBoost | Royston-Parmar | XGBoost |
| --- | --- | --- | --- | --- |
| Prediction months | 12 | | 6 | |
| Training size | 8458 ± 109 | | 8782 ± 116 | |
| Test size | 2113 ± 109 | | 2194 ± 116 | |
| % died | 61.6 ± 1.9 | | 20.3 ± 1.1 | |
| AUC | 0.798 ± 0.021 | 0.819 ± 0.011 | 0.768 ± 0.01 | 0.782 ± 0.004 |
| AUPRC | 0.449 ± 0.016 | 0.857 ± 0.017 | 0.127 ± 0.008 | 0.474 ± 0.014 |
| Accuracy | 0.732 ± 0.023 | 0.749 ± 0.015 | 0.661 ± 0.02 | 0.727 ± 0.022 |
| Sensitivity | 76.2 ± 3.3 | 77.1 ± 2.3 | 76.1 ± 3.6 | 64.8 ± 3.8 |
| Specificity | 68.5 ± 2.1 | 71.2 ± 2.5 | 63.6 ± 3.2 | 74.7 ± 3.7 |
| F1 | 0.778 ± 0.025 | 0.790 ± 0.018 | 0.476 ± 0.019 | 0.491 ± 0.011 |
| PPV | 79.5 ± 2.2 | 81.1 ± 1.7 | 34.7 ± 2.1 | 0.397 ± 2.1 |
| NPV | 64.3 ± 2.8 | 66.0 ± 1.2 | 91.3 ± 1.1 | 0.893 ± 0.7 |

**Supp. Table 7** Top 15 features important for 12 and 6-month mortality prediction​​ for RP models and their association with mortality risk

| **Rank** | **Feature** | **Association with mortality risk (higher/lower risk)** |
| --- | --- | --- |
| 1 | Age | Higher |
| 2 | BMI | Lower |
| 3 | Albumin | Lower |
| 4 | Bicarbonate | Higher |
| 5 | Hemoglobin | Lower |
| 6 | Limb Onset | Lower |
| 7 | FVC | Lower |
| 8 | Hematocrit | Lower |
| 9 | Chloride | Lower |
| 10 | Bulbar Onset | Higher |
| 11 | Pulse | Higher |
| 12 | Sodium | Higher |
| 13 | Abs. Basophil | Lower |
| 14 | Creatinine | Higher |
| 15 | ALSFRS-R | Lower |

**Supp. Table 8** Mortality prediction performance using XGBoost and various observation window lengths

| Observation months | Single visit | 2 | 3 | Single visit | 2 | 3 |
| --- | --- | --- | --- | --- | --- | --- |
| Prediction months |  | 12 |  |  | 6 |  |
| Training size | 5081 ± 75 | | |  | 5338 ± 77 |  |
| Test size | 1269 ± 77 | | |  | 1334 ± 77 |  |
| % died | 55.3 | 66.7 | 71.7 | 7.0 | 22.1 | 29.7 |
| AUC | 0.763 ± 0.015 | 0.836 ± 0.017 | 0.865 ± 0.018 | 0.788 ± 0.016 | 0.772 ± 0.021 | 0.777 ± 0.018 |
| AUPRC | 0.766 ± 0.022 | 0.894 ± 0.014 | 0.930 ± 0.007 | 0.225 ± 0.021 | 0.463 ± 0.031 | 0.568 ± 0.035 |
| Accuracy | 69.7 ± 1.2 | 78.4 ± 1.5 | 81.7 ± 1.9 | 81.3 ± 4.2 | 73.9 ± 1.0 | 71.9 ± 1.5 |
| Sensitivity | 71.3 ± 1.7 | 83.3 ± 2.9 | 87.7 ± 5.1 | 50.6 ± 12.1 | 55.0 ± 7.6 | 59.3 ± 4.8 |
| Specificity | 67.7 ± 2.1 | 68.7 ± 5.3 | 66.9 ± 8.9 | 83.6 ± 5.4 | 79.3 ± 1.1 | 77.3 ± 3.6 |
| F1 | 0.731 ± 0.013 | 0.843 ± 0.011 | 0.872 ± 0.014 | 0.274 ± 0.013 | 0.480 ± 0.043 | 0.556 ± 0.021 |
| PPV | 73.1 ± 2.3 | 84.3 ± 2.3 | 87.2 ± 2.8 | 19.3 ± 2.6 | 42.8 ± 2.6 | 52.7 ± 4.1 |
| NPV | 65.6 ± 2.3 | 67.4 ± 4.4 | 69.2 ± 9.9 | 95.8 ± 1.1 | 86.2 ± 2.1 | 81.8 ± 1.9 |

**Supp. Table 9** 1-year mortality prediction performance for mildly and severely affected patients

| Observation months | Single visit | | 2 | | 3 | |
| --- | --- | --- | --- | --- | --- | --- |
| Mild/Severe  % | Mild  59.7 ± 4.7 | Severe  40.3 ± 4.7 | Mild  56.6 ± 4.8 | Severe  43.4 ± 4.8 | Mild  54.4 ± 5.3 | Severe  45.6 ± 5.3 |
| % died | 49.4 ± 3.2 | 63.7 ± 2.1 | 60.1 ± 4.3 | 74.7 ± 2.4 | 65.1 ± 4.1 | 79.2 ± 2.4 |
| AUC | 0.771 ± 0.016 | 0.725 ± 0.036 | 0.841 ± 0.029 | 0.811 ± 0.024 | 0.865 ± 0.031 | 0.848 ± 0.028 |
| AUPRC | 0.736 ± 0.018 | 0.794 ± 0.034 | 0.871 ± 0.032 | 0.912 ± 0.014 | 0.905 ± 0.029 | 0.947 ± 0.008 |
| Accuracy | 68.8 ± 1.4 | 70.8 ± 2.5 | 76.8 ± 2.3 | 80.2 ± 2.6 | 79.6 ± 2.7 | 84.1 ± 2.0 |
| Sensitivity | 62.6 ± 2.5 | 81.4 ± 2.0 | 79.2 ± 3.1 | 87.7 ± 2.3 | 84.6 ± 6.4 | 90.8 ± 3.9 |
| Specificity | 74.9 ± 1.6 | 52.2 ± 5.6 | 73.8 ± 6.7 | 58.0 ± 6.5 | 71.3 ± 8.4 | 57.8 ± 11.5 |
| F1 | 0.664 ± 0.018 | 0.780 ± 0.021 | 0.804 ± 0.019 | 0.869 ± 0.020 | 0.843 ± 0.022 | 0.900 ± 0.016 |
| PPV | 70.8 ± 3.0 | 74.9 ± 3.1 | 82.0 ± 5.4 | 86.1 ± 2.1 | 84.5 ± 5.1 | 89.3 ± 2.0 |
| NPV | 67.2 ± 3.6 | 61.5 ± 3.1 | 70.0 ± 5.5 | 61.5 ± 4.8 | 71.7 ± 10.9 | 63.5 ± 8.6 |

**Supp. Table 10** Mortality prediction using the Top 7 features on PRO-ACT Dataset

| Model | Royston-Parmar | XGBoost | Royston-Parmar | XGBoost |
| --- | --- | --- | --- | --- |
| Prediction months | 12 | | 6 | |
| Training size | 8458 ± 109 | | 8782 ± 116 | |
| Test size | 2113 ± 109 | | 2194 ± 116 | |
| % died | 61.6 | | 20.3 | |
| AUC | 0.751 ± 0.019 | 0.798 ± 0.011 | 0.719 ± 0.012 | 0.748 ± 0.007 |
| AUPRC | 0.466 ± 0.018 | 0.842 ± 0.019 | 0.134 ± 0.007 | 0.408 ± 0.018 |
| Accuracy | 0.699 ± 0.015 | 0.73 ± 0.012 | 0.609 ± 0.039 | 0.681 ± 0.014 |
| Sensitivity | 73.3 ± 2.5 | 75.3 ± 2.6 | 73.9 ± 7.5 | 68.2 ± 3.7 |
| Specificity | 64.2 ± 1.5 | 69.2 ± 2.3 | 57.4 ± 7.1 | 68.0 ± 2.4 |
| F1 | 0.750 ± 0.019 | 0.774 ± 0.016 | 0.434 ± 0.015 | 0.464 ± 0.022 |
| PPV | 76.7 ± 1.7 | 79.7 ± 0.5 | 30.8 ± 0.9 | 35.2 ± 1.8 |
| NPV | 60.1 ± 1.5 | 63.7 ± 2.0 | 89.9 ± 1.1 | 89.4 ± 0.2 |

**Supp. Table 11** Mortality prediction on the Celecoxib trial using all features

| Model | Royston-Parmar | XGBoost | Royston-Parmar | XGBoost |
| --- | --- | --- | --- | --- |
| Prediction months | 12 | | 6 | |
| Test size | 329 | | 915 | |
| % died | 67.2 | | 12.3 | |
| AUC | 0.866 ± 0.009 | 0.840 ± 0.007 | 0.894 ± 0.006 | 0.907 ± 0.004 |
| AUPRC | 0.483 ± 0.003 | 0.918 ± 0.004 | 0.068 ± 0 | 0.607 ± 0.013 |
| Accuracy | 0.764 ± 0.023 | 72.0 ± 1.0 | 0.855 ± 0.012 | 88.7 ± 0.8 |
| Sensitivity | 70.8 ± 3.3 | 64.3 ± 1.4 | 69.8 ± 2.5 | 55.4 ± 4.4 |
| Specificity | 87.8 ± 1.0 | 87.8 ± 1.2 | 87.8 ± 1.4 | 93.5 ± 1.6 |
| F1 | 0.800 ± 0.023 | 0.756 ± 0.010 | 0.546 ± 0.022 | 0.550 ± 0.009 |
| PPV | 92.2 ± 0.7 | 91.5 ± 0.8 | 45.0 ± 2.8 | 55.1 ± 4.2 |
| NPV | 59.6 ± 2.7 | 54.6 ± 1.0 | 95.3 ± 0.4 | 93.7 ± 0.5 |

**Supp. Table 12** Mortality prediction on the SG ALS Clinical Database using all features

| Model | Royston-Parmar | XGBoost | Royston-Parmar | XGBoost |
| --- | --- | --- | --- | --- |
| Prediction months |  | 12 |  | 6 |
| Test size | 344 | | 344 | |
| % died | 9.9 | | 3.5 | |
| AUC | 0.714 ± 0.018 | 0.768 ± 0.02 | 0.748 ± 0.024 | 0.753 ± 0.022 |
| AUPRC | 0.064 ± 0.001 | 0.222 ± 0.017 | 0.021 ± 0.001 | 0.11 ± 0.024 |
| Accuracy | 0.565 ± 0.036 | 0.664 ± 0.033 | 0.642 ± 0.03 | 0.772 ± 0.023 |
| Sensitivity | 78.2 ± 6.8 | 74.7 ± 4.5 | 66.7 ± 11.8 | 45.0 ± 4.6 |
| Specificity | 54.1 ± 4.6 | 65.5 ± 3.6 | 64.2 ± 3.4 | 78.3 ± 2.3 |
| F1 | 0.262 ± 0.014 | 0.306 ± 0.025 | 0.115 ± 0.013 | 0.122 ± 0.016 |
| PPV | 15.8 ± 0.9 | 19.3 ± 1.8 | 6.3 ± 0.7 | 7.0 ± 1.0 |
| NPV | 95.8 ± 1.0 | 95.9 ± 0.7 | 98.2 ± 0.6 | 97.5 ± 0.2 |

**Supp. Table 13** Mortality prediction on the Celecoxib trial using top 7 features

| Model | Royston-Parmar | XGBoost | Royston-Parmar | XGBoost |
| --- | --- | --- | --- | --- |
| Prediction months | 12 | | 6 | |
| Test size | 329 | | 915 | |
| % died | 67.2 | | 12.3 | |
| AUC | 0.808 ± 0.01 | 0.793 ± 0.011 | 0.865 ± 0.003 | 0.851 ± 0.006 |
| AUPRC | 0.509 ± 0.004 | 0.882 ± 0.008 | 0.07 ± 0 | 0.489 ± 0.022 |
| Accuracy | 0.707 ± 0.009 | 64.4 ± 1.8 | 0.817 ± 0.034 | 84.6 ± 1.0 |
| Sensitivity | 63.5 ± 2.0 | 51.9 ± 2.9 | 72.8 ± 05.9 | 51.2 ± 4.0 |
| Specificity | 85.4 ± 1.5 | 90.0 ± 1.8 | 83.0 ± 4.7 | 89.3 ± 1.6 |
| F1 | 0.744 ± 0.012 | 0.662 ± 0.023 | 0.501 ± 0.027 | 0.453 ± 0.016 |
| PPV | 89.9 ± 0.7 | 91.4 ± 1.3 | 38.6 ± 04.7 | 40.7 ± 2.3 |
| NPV | 53.4 ± 1.0 | 47.8 ± 1.5 | 95.6 ± 0.7 | 92.8 ± 0.4 |

**Supp. Table 14** Mortality prediction on SG ALS Clinical Dataset using the Top 7 features

| Model | Royston-Parmar | XGBoost | Royston-Parmar | XGBoost |
| --- | --- | --- | --- | --- |
| Prediction months | 12 | | 6 | |
| Test size | 344 | | 344 | |
| % died | 9.9 | | 3.5 | |
| AUC | 0.707 ± 0.006 | 0.681 ± 0.028 | 0.744 ± 0.014 | 0.706 ± 0.045 |
| AUPRC | 0.064 ± 0 | 0.15 ± 0.01 | 0.022 ± 0 | 0.07 ± 0.013 |
| Accuracy | 0.563 ± 0.023 | 0.711 ± 0.019 | 0.615 ± 0.068 | 0.784 ± 0.025 |
| Sensitivity | 80.6 ± 4.5 | 52.4 ± 9.6 | 75.0 ± 5.9 | 40.0 ± 14.9 |
| Specificity | 53.7 ± 2.7 | 73.2 ± 2.6 | 61.0 ± 7.2 | 79.8 ± 2.7 |
| F1 | 0.267 ± 0.013 | 0.263 ± 0.033 | 0.121 ± 0.011 | 0.114 ± 0.036 |
| PPV | 16.0 ± 0.8 | 17.6 ± 2.0 | 6.6 ± 0.7 | 06.6 ± 2.1 |
| NPV | 96.2 ± 0.8 | 93.4 ± 1.2 | 98.6 ± 0.2 | 97.4 ± 0.6 |
